# A Comparative Evaluation of Powder Characteristics of Recycled Material from Bronze Grinding Chips for Additive Manufacturing

**DOI:** 10.3390/ma17143396

**Published:** 2024-07-09

**Authors:** Eckart Uhlmann, Julian Polte, Janek Maria Fasselt, Vinzenz Müller, Christian Klötzer-Freese, Rafael Kleba-Ehrhardt, Max Biegler, Michael Rethmeier

**Affiliations:** 1 Fraunhofer Institute for Production Systems and Design Technology IPK, 10587 Berlin, Germany; eckart.uhlmann@ipk.fraunhofer.de (E.U.); julian.polte@ipk.fraunhofer.de (J.P.); vinzenz.mueller@ipk.fraunhofer.de (V.M.); max.biegler@ipk.fraunhofer.de (M.B.); michael.rethmeier@bam.de (M.R.); 2 Machine Tools and Production Engineering, Institute for Machine Tools and Factory Management IWF, Technische Universität Berlin, 10587 Berlin, Germany; 3 Mecklenburger Metallguss GmbH, 17192 Waren, Germany; c.kloetzer@mmg-propeller.de; 4 Advanced Ceramic Materials, Institute of Material Science and Technology, Technische Universität Berlin, 10623 Berlin, Germany; r.kleba-ehrhardt@ceramics.tu-berlin.de; 5 Federal Institute for Materials Research and Testing BAM, 12205 Berlin, Germany

**Keywords:** grinding chips, comminution, aluminium bronze, additive manufacturing, recycling, sustainability

## Abstract

In the manufacturing process of ship propellers, large quantities of grinding chips are generated. These grinding chips result from the finishing of the blade surfaces after the primary casting process of the propeller. The aim of this study was to investigate and compare different preparation processes used to produce chip powders with sufficient powder quality for the additive manufacturing process of directed energy deposition. The preparation of the samples was performed through different sieving, milling and re-melting processes. For the characterization of the prepared samples, powder analysis according to relevant industry standards was carried out. It was found that the re-melting processes result in superior powder quality for additive manufacturing in terms of particle size, morphology, and flowability. For some characteristics, the powder exhibits even better properties than those of commercial powders. Furthermore, the powder properties of the milled samples demonstrate a promising potential for use in additive manufacturing.

## 1. Introduction

The manufacturing of large cast components often involves multiple machining and grinding steps, generating machining waste and scrap. In the case of ship propeller casting, the propeller blades are ground to achieve the final blade shape. These components are typically composed of nickel aluminium bronze (NAB) CuAl10Ni5Fe5, a popular alloy for maritime applications due to its good mechanical properties and high corrosion resistance in sea water [1]. The grinding of these components generates a substantial amount of waste in the form of chips, often amounting to several hundred kilograms per part. The low value of these waste chips and the costly, multi-step process of re-melting for casting make their reuse a concern. In this context, this study investigates different approaches to upcycle NAB grinding chips to additive manufacturing (AM) feedstock for a powder- and laser-based directed energy deposition (DED-LB) process, aiming to facilitate cost-effective and locally sourced production of recycled powder and promote sustainability in AM and the maritime sector. 

In the context of AM, the quality of metal powder used as feedstock is crucial for both the manufacturing process and the final part quality. To assess AM powders, various characterization methods can be employed and typically involve analyzing the powder’s particle size distribution (PSD), particle morphology, flow properties, and chemical composition. Research in the literature explores suitable techniques and procedures for this characterization. For instance, Zegzulka et al. [2] extensively characterized various metal powders. Wimler et al. [3] and Mitterlehner et al. [4] have undertaken investigations into distinct methodologies for quantifying the PSD and morphological attributes of AM powders. Kiani et al. [5] employed statistical techniques to describe the characteristics of AM powders. Studies carried out by Baesso et al. [6] and Spierings et al. [7] encompassed the utilization and comparison of standardized assessments designed to determine the flow characteristics of powder materials.

There are multiple studies that investigated the use of metal chips as feedstock for AM processes. While some authors, such as Mahmood et al. [8], directly used chips as feedstock, most studies applied mechanical comminution to improve chip powder properties. For example, Afshari et al. [9] compared jet milling to ball milling for copper bronze (CuSn10) chip preparation. In ball milling, grinding balls collide with the material, breaking it down into smaller particles. In jet milling, high-velocity gas streams are used to propel solid particles into a grinding chamber or nozzles where they collide and fracture into smaller sizes. Jet milling resulted in an irregular-shaped, low-oxidation powder with a minimal hardness increase, while ball milling produced nearly round particles with higher oxygen content (O) and increased hardness.

Jackson et al. [10] produced specimens from ball-milled 316L steel chips. The authors measured foreign titanium (Ti) and high oxygen (O_2_) content in the specimens and traced back the titanium to the coating of the milling tool used during machining. The oxygen was likely added during ball milling.

Fullenwider et al. [11] used a two-stage ball milling process to prepare 304L steel chips for AM. Large balls were used for coarse comminution, followed by small balls for spherical shaping. The resulting powder had nearly spherical particles and particle sizes in the range of 38 µm ≤ X_Fmin_ ≤ 150 µm. The authors created weld tracks using a DED-LB system, although it is important to clarify that the material was not deposited during the welding process but was instead applied to the substrate in advance. Dhiman et al. [12] came to a similar conclusion as Fullenwidder et al. [11] by investigating the preparation of chips of a titanium alloy (Ti6Al4V) for the powder-bed fusion AM process using a two-stage ball mill approach. A particle size distribution of 50 µm ≤ X_Fmin_ ≤ 150 µm and a suitable spherical shape was achieved with a ball milling time of t_m_ = 18 h.

Besides comminution, re-melting of chips can be used to change their shape. For example, Razumov et al. [13] generated metal powder by melting steel machining chips with a plasma torch in a Tekna plasma system. The production of spherical particles with an acceptable PSD for a laser powder-bed fusion process was achieved. Additionally, atomization can be used to produce powders from chips. While gas atomization is the most common method to produce AM powders [14], ultrasonic atomization can be suitable when the qualitative demand is high and the quantitative demand is low. In ultrasonic atomization, the material is melted down and dropped on a sonotrode that vibrates with frequencies f in the kHz-range. As a result, fine metal droplets are ejected. Due to their surface tension, they can be observed to form into a spherical shape in which they solidify. The process takes place in an inert atmosphere of argon to prevent oxidation. The ultrasonic atomization approach was initially described by Lierke and Grießhammer [15] in 1967. More recently, Żrodowski et al. [16] investigated ultrasonic atomization to produce AM powders with good results in regards to morphology, PSD, and flow properties. In a previous study from Müller et al. [17] and the authors of this study used ultrasonic atomized metal powder produced from the same grinding chips investigated in this paper to produce DED-LB specimens.

The studies indicate challenges in recycling waste to feedstock, including issues like particle morphology, particle sphericity, PSD, contaminations from the manufacturing process as well as oxidation of the chips. This paper applies three methods to produce AM powders from NAB chips generated during ship propeller manufacturing:

- One-stage ball milling;
- Impact whirl milling;
- Ultrasonic atomization.

The powder characteristics of the “chip powders” in correlation to the different upcycling methods are described, and the results are evaluated following the German AM guideline VDI 3405 Sheet 2.3 [18] to determine the suitability for using the material as feedstock for a DED-LB process.

## 2. Materials and Methods

### 2.1. Base Material and Reference Powder

**Figure 1:**
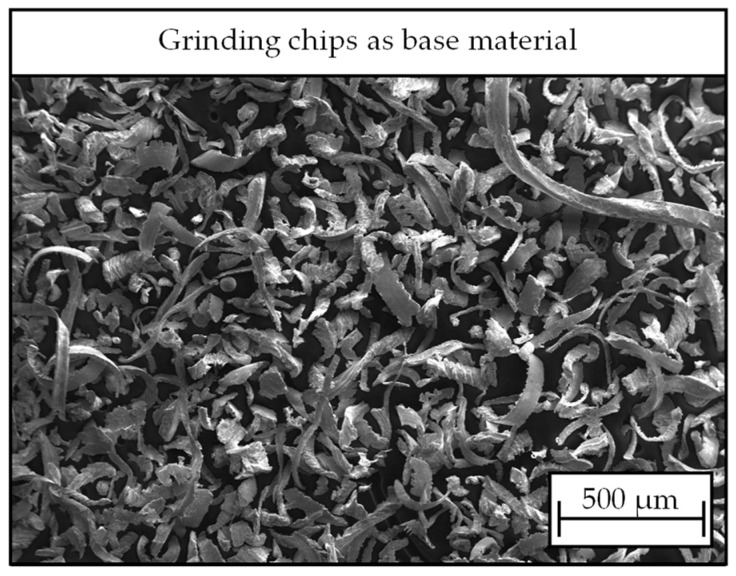
SEM image of base material used to produce chip powders in this study.

The base material is nickel aluminium bronze CuAl10Ni5Fe5 (CC333G). Its alloy composition is specified in EN 1982 [19]. The material is characterized by high strength, corrosion resistance in seawater, and excellent fatigue properties. It has good castability and is widely used in the marine sector for ship propellers, pump housings, or marine propeller shafts. The chips used as a base material in this study were collected after the grinding process of ship propeller production at Mecklenburger Metallguss GmbH, Waren, Germany. Figure 1 shows an SEM image of the material. Due to the high cohesion and unfavorable particle size distribution for certain analyses carried out for this study, the raw material was sieved with an analysis sieve of mesh width d_mesh_ = 125 µm.

To obtain reference values for powder properties, commercially available gas-atomized (GA) metal powder from aluminium bronze CuAl9.5Fe (Oerlikon Metco 51NS, Oerlikon Metco AG, Wohlen, Switzerland) was characterized and used to compare chip powder properties.

### 2.2. Powder Processing

Table 1 lists all investigated materials from this study with their alloy composition and processing information. 

Ball-milled powders were produced in two batches by LITech GmbH, Sankt Andrä, Austria. The first sample (BM-1) was ground at a duration of t_mill_ = 8 h, the second sample (BM-2) at t_mill_ = 12 h. Ball milling balls made of 100Cr6 steel was used as grinding media. No inert gas atmosphere was used and the ball-milled powders were not sieved after milling.

Impact whirl milling was performed on a DemiNo 2250 by Aufbereitungstechnologie Noll GmbH, Bobingen, Germany. Two samples with a mill rotation speed of n_mill_ = 3000 rpm (IWM-1) and n_mill_ = 8000 rpm (IWM-2) were produced. Nitrogen was used as the inert gas during milling to reduce oxidation. The resulting chip powders were sieved with a d_m_ = 150 µm mesh sieve during the milling process. Particles bigger than X_Fmin_ = 150 µm were fed back into the mill while smaller particles were collected. Subsequently, fine particles with X_Fmin_ < 60 µm were sieved out as well. 

Ultrasonic atomization was employed on an AUS 500 Atomizer from the company AMAZEMET Sp. z o.o., Warsaw, Poland, and BluePower Casting Systems GmbH, Walzbachtal, Germany, to atomize the aluminium bronze chips into powder. In the process, the feedstock was heated to ϑ = 1300 °C in an induction furnace and fed through a nozzle system with a system pressure of p = 1.5 bar onto a carbon fiber sonotrode vibrating at a frequency of f = 40 kHz. An inert gas atmosphere of argon with grade 5.0 was used for both melting and atomization. After atomization, the powder was sieved with a d_m_ = 200 µm mesh sieve to remove spatters.

### 2.3. Powder Analysis

The determination of the chemical composition was carried out by the means of energy dispersive X-ray analysis (EDX) with a detector (Si(Li), <129 eV) from Remx GmbH, Bruchsal, Germany. Scanning electron microscopy was performed on a LEO 1455 VP from the company ZEISS GmbH, Oberkochen, Germany.

The PSD as well as morphology properties were measured on the basis of ISO 13322-2 [20] via dynamic image analysis on a CamSizer X2 from the company Microtrac Retsch GmbH, Haan, Germany.

The flowability of the materials was assessed using various methods, including the Carney flowmeter, the Hausner ratio, determined from bulk and tapped density measurements, as well as the angle of repose (AOR). Three measurements were made for each characterization method.

The Carney flow time measurement determines the discharge time a powder needs to flow through a funnel and was performed according to ASTM B964 on the Carney flowmeter from the company LPW Technologies, Cheshire, UK, with an orifice diameter of d_o_ = 5 mm [21]. The advantage of the Carney flowmeter over the Hall or Gustafson flowmeter is that the comparatively larger outlet allows the measurement of more cohesive powders.

The determination of the powder density *ρ*_P_ was carried out based on images of powder cross-sections taken with an optical microscope, Infinite Focus, from the company Alicona Imaging GmbH, Raaba, Austria.

The bulk density *ρ*_Sch_ was determined after ISO 3923 [22] using a PT-SV100 Scottvolumeter from the company Pharma Test Apparatebau AG, Hainburg, Germany, and indicates the density of the powder in the loose uncompressed state. For the bulk density *ρ*_Sch_ test, m_p_ = 130 g of powder was used, which was passed through a sieve and several inhibition plates in the PT-SV100 into a standardized measuring cup with a volume of V = 25 cm^3^. Excess powder on the measuring cup was scraped off using a blade. The bulk density *ρ*_Sch_ was calculated over the volume V and the determined weight m_b_ of the cup content. The tap density *ρ*_K_ indicates the density of a powder in a compressed state and was measured using a STAV 2003 from the company Engelsmann AG, Ludwigshafen, Germany, according to ISO 3953 [23]. A measuring cylinder with a volume of V_c_ = 25 cm^3^ was used for the measurements, which was filled to a volume of V_cf_ = 20 cm^3^ with powder. After n = 3000 set taps, the compressed volume was read and the tapped density was calculated.

From the ratio of bulk and tapped density, the Hausner ratio was calculated. The ratio serves as an index for flowability. Powders with a Hausner ratio close to one can be considered more flowable, while powders with a higher Hausner ratio can be considered more cohesive [24].

The AOR was determined according to ISO 4324 [25]. For the formation of the powder cone, V_cf_ = 150 mL of powder was carefully poured through the funnel with a defined orifice of d_o_ = 10 mm from the company LPW Technologies, Cheshire, UK. The powder cone builds up on a round plate with a set diameter located under the funnel. The AOR was calculated from the subsequent measurement of the powder cone.

## 3. Results and Discussion

In order to evaluate the suitability of the recycled grinding chips for AM, the powder and material properties were determined using selected powder characterization methods following the guideline VDI 3405 Sheet 2.3.

### 3.1. Particle Size Distribution

**Figure 2:**
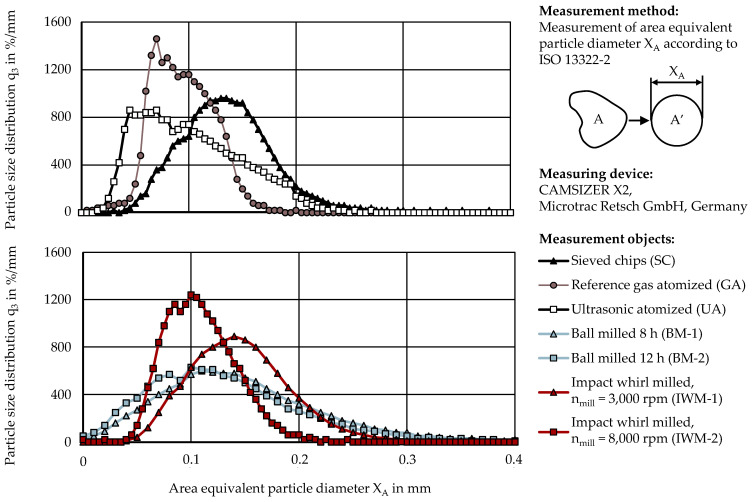
Particle size distributions q_3_ of area equivalent particle diameter X_A_. The plots are split for better readability.

Measurements of PSD and morphology were carried out via dynamic image analysis. In two split plots, Figure 2 shows the PSD with the area-equivalent particle diameter X_A_ for the investigated powders of this study. The PSD of metal powders for powder-based DED processes usually lies in the range of X_Fmin,10_ = 50 µm to X_Fmin,90_ = 150 µm [26], while some commercially available powders also have wider ranges in PSD with X_Fmin,90_ > 200 µm. The gas-atomized reference powder in this study had a PSD of X_A,10_ = 64 µm to X_A,90_ = 135 µm. 

The PSDs for the ball-milled materials BM-1 (X_A,10_ = 66 µm to X_A,90_ = 254 µm) and BM-2 (X_A,10_ = 54 µm to X_A,90_ = 235 µm) did not differ significantly from each other despite the increased milling time of 50% for BM-2, indicating a decreased margin utility of grinding time with the given milling parameters. While not done for this study, a possible approach to further decrease particle size faster could be to use a two-stage ball milling process as done by Fullenwider et al. [11]. The ball milling curves of the PSD showed by far the widest distribution under the produced chip powders. However, it must be noted that the ball-milled powders were not subject to sieving after milling.

The materials produced via impact whirl milling were sieved during and after the process (d_mesh_ = 60 µm and d_mesh_ = 150 µm sieve) to gain the maximum material yield. Therefore, the sieved chips (SC) presented a good baseline for comparisons as they were screened with a d_mesh_ = 125 µm analysis sieve. The PSD displayed in Table 2 for IWM-1 (X_A,10_ = 94 µm to X_A,90_ = 212 µm) did not differ significantly from the untreated material SC (X_A,10_ = 81 µm to X_A,90_ = 188 µm), indicating that the slower rotation speed of n_mill_ = 3000 rpm was not sufficient to have a clear effect on the PSD. At a speed of n_mill_ = 8000 rpm, the number of fine particles notably increased and the particles became shorter, and the PSD for IWM-2 (X_A,10_ = 72 µm to X_A,90_ = 155 µm) became closer to the gas-atomized reference material. The material produced by ultrasonic atomization had a relatively wide range of PSD (X_A,10_ = 48 µm to X_A,90_ = 174 µm). Compared to the other materials, the fine content of particles was highest in the ultrasonic atomized material.

### 3.2. Particle Shape (Morphology)

For AM feedstock, particle morphology is a critical property that determines powder quality, flowability, and ultimately process stability and part quality. For powder-bed fusion processes, the particle morphology is mainly relevant to ensure good spreadability and a homogeneous powder layer [2]. For powder-based DED processes, the flowability of the material is important to ensure robust powder deposition [27].

Particle morphology was quantitively analyzed via digital image analysis comparing the aspect ratio AR, which is calculated as a ratio of the shortest (X_Fmin_) and longest (X_Fmax_) Feret length of a particle using the following formula:

AR = X_Fmin_/X_Fmax_ (1)

Qualitative and image-based morphology properties were obtained using SEM images of the materials. Expectedly, the particle morphologies of the individual powder samples in this study differed greatly from each other and were characteristic for the corresponding preparation processes. Table 2 shows the mean values for AR as well as the percentage of almost-spherical particles with AR > 0.9.

**Figure 3:**
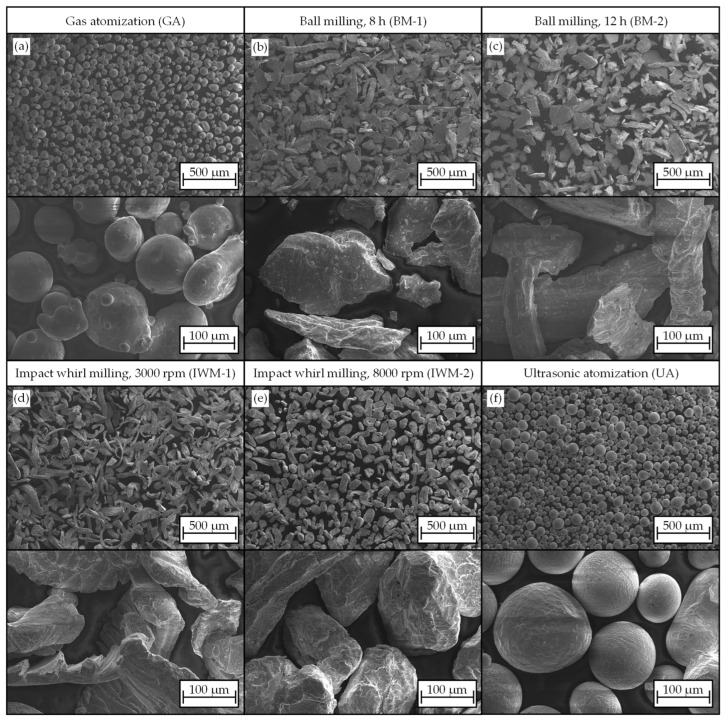
Overview of SEM images showing the materials of this study: Gas atomized reference powder (**a**), ball milled chips after t_mill_ = 8 h (**b**) and t_mill_ = 12 h (**c**), chips from the impact whirl mill at n_mill_ = 3000 rpm (**d**) and n_mill_ = 8000 rpm (**e**), powder produced via ultrasonic atomization (**f**).

One primary reason for the prevalent use of a gas-atomized powder as feedstock in AM is the resulting high sphericity of the powders [2]. Concerning the morphology, the GA material exhibited expected outcomes, with a mean aspect ratio AR_mean_ = 0.843 that is close to AR_mean_ = 1. The SEM images validate the majority presence of predominantly spherical particles in the reference powder (Figure 3a). However, the images also revealed a noticeable number of satellites, agglomerations, and elongated particles, contributing to the observation that, despite the high mean aspect ratio, only 43.5% of particles can be categorized as “perfectly” spherical.

For the powders processed by mechanical comminution, the SEM images still reveal a more or less clear chip structure (Figure 3b,c,e,f). Both ball-milled samples BM-1 and BM-2 had a similar aspect ratio of AR_mean_ = 0.56 and AR_mean_ = 0.58, respectively. Compared to the starting material, the particles displayed reduced fragmentation with evident signs of breakage and rupture on the surfaces. A fine fraction formed, which increased with extended milling time t_f_. Cold welding, as observed by some authors during ball milling, did not occur [28]. Despite the difference in milling time, the two samples did not show a significant deviation in their morphology. 

The SEM image of the IWM-1 particles from the impact mill also shows a distinct chip shape (Figure 3c,f). With an aspect ratio of AR_mean_ = 0.54 and visual similarities, the material did not significantly differ from the raw chips. The rotational speed of n_mill_ = 3000 rpm used to process IWM-1 appears insufficient to notably alter the particles. Conversely, IWM-2 showed a clear rounding of the grains and a more homogenous distribution. The proportion of “perfectly” spherical particles (AR > 0.9) increased from 1.9% (IWM-1) to 4.9% (WM-2). The impact whirl milling process did not yield a significant fraction of fines, as observed in ball milling.

Sample UA, depicted in Figure 3d, showed a predominant formation of spherical particles. As anticipated due to the complete re-melting in the ultrasonic atomization process, chip shapes were no longer present. A limited number of satellite grains and agglomerations formed on the particles as with the reference powder due to the atomization process. Nevertheless, sample UA had the highest mean aspect ratio of AR_mean_ = 0.94 and a superior share of 82% of particles with AR > 0.9. In comparison to the reference sample GA, there was a substantially lower amount of satellite grains and agglomerations.

In summary, the SEM images illustrate the distinct differences in particle morphology among all the powders of this study. The ball-milled powders displayed a reduced particle size without significant rounding and a fine fraction. While conventionally manufactured powder GA and sample UA share similarities in shape, the sphericity of UA powder was significantly higher. Powder IWM-1 showed no apparent change in morphology compared to the raw material, while sample IWM-2 exhibited a more pronounced rounding of the chips with increased impact mill speed.

### 3.3. Determination of Powder Density

**Figure 4:**
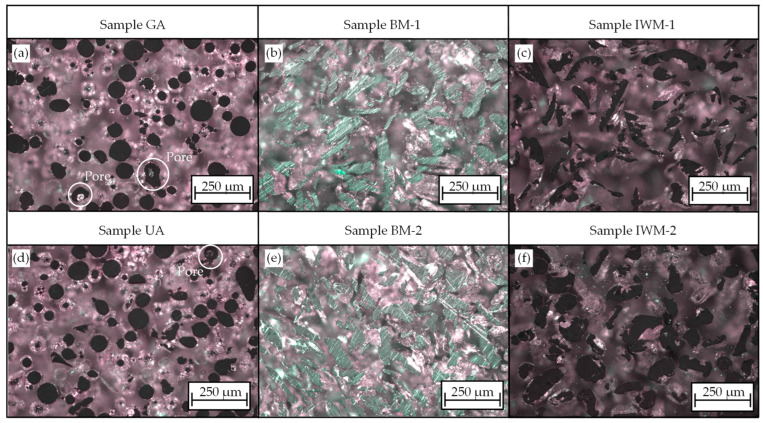
Light microscope images illustrating powder density and cavities in chip powders for gas atomized material (**a**), ball milled materials (**b**,**c**), ultrasonic atomized powder (**d**) and chips produced via impact whirl milling (**e**,**f**).

Metallographic cross-sections of the investigated powders can be seen in Figure 4. Expectedly, no inclusions or enclosed areas could be detected for samples BM-1 and BM-2, as these consist of broken chip fractures. Figure 4f shows that in sample IWM-2, the chips were not only crushed during impact whirl milling but also rolled up. As a result, some particles have cavities, which, similar to the pores in atomized powders, may have a negative effect on the density and component quality. Sample UA exhibited almost no gas inclusions, whereas the commercially processed powder GA showed more gas pores. 

### 3.4. Chemical Composition

The chemical composition of AM feedstocks plays a crucial role in determining material quality and part properties. The results of the EDX analyses for all base materials are shown in Table 3 together with the standard composition of the base material alloy CC333G as defined in DIN 198 [19]. Besides some minor deviations, all analyzed samples obtained from grinding chips were well within the range of mass fractions defined by the standard. The composition of the gas-atomized material also met the theoretical composition. While EDX analysis is a good tool to qualitatively determine the elements, the technology does not generally allow the gathering of precise compositions [29]. Against this backdrop, the low amount of measured aluminium in sample BM-2 is considered a measurement deviation during EDX analysis. Żrodowski et al. [16] detected a depletion of elements like manganese in certain alloys processed via ultrasonic atomization. However, according to the EDX analysis, manganese content in material UA was the highest, and the chemical composition of material UA did not differ substantially from the other samples. It can be stated that the base material’s chemical composition in all samples was not altered significantly by any of the investigated recycling processes.

### 3.5. Contaminations and Impurities

In the context of recycled materials as investigated in this study, the consideration of the composition of the base material extends to potential contaminations originating from the grinding process as well as from the recycling processes. In a previous study by Müller et al. [30,31] that focused on the same raw aluminium bronze chips, abrasive particles from the grinding belts were identified in the chips.

**Figure 5:**
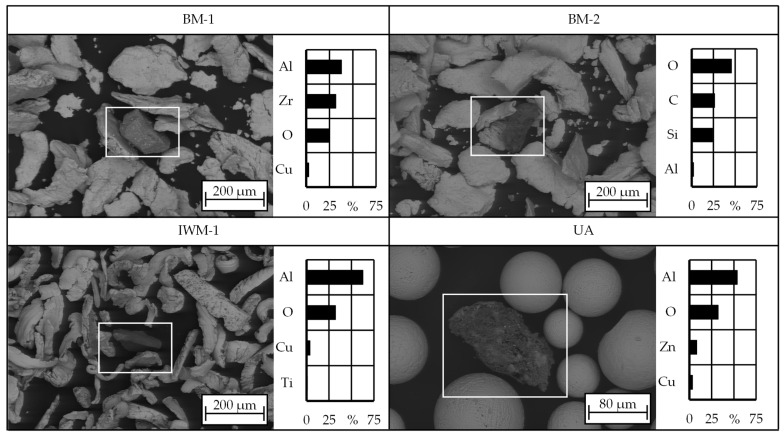
Exemplary SEM images of ceramic impurities found in BM-1, BM-2, IWM-1 and UA.

Therefore, SEM and EDX were used to find and identify foreign particles in the chip powders. All manufactured powder samples contained ceramic contaminants consisting of either zirconium oxide (ZrO_2_), aluminum oxide (Al_2_O_3_) or silicon dioxide (SiO_2_). All three candidates are commonly used as grinding abrasives and are impurities from the ship propeller manufacturing process. Figure 5 shows exemplary SEM images together with the chemical composition of the respective foreign particles. While only a few such impurities were detected in the UA material, the SEM analysis revealed significantly higher quantities of ceramic particles in the powders from mechanical comminution ball milling and impact whirl milling. This type of contamination is critical for an AM process as it impairs the integrity of the built-up structure. It can be assumed that the particles would be found as inclusions in the additive structures due to the higher melting point, where they contribute to reducing the mechanical properties, for instance, as crack initiators.

**Figure 6:**
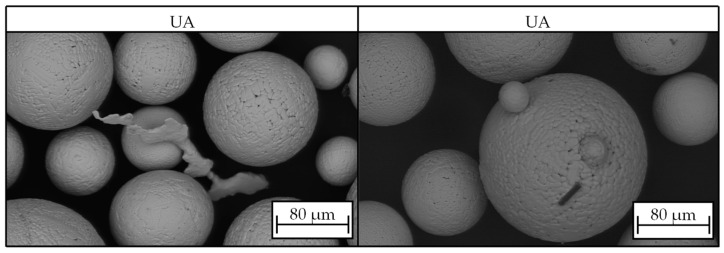
SEM images of particles from sample UA with spatters and carbon fiber.

In addition, SEM images of material UA showed single spatters and fine carbon fibers of X_Fmax_ ≈ 20 µm length (Figure 6). These fibers most likely came from the vibrating sonotrode that was employed in the ultrasonic atomization process. The sonotrode was made from binder-free woven carbon fibers. Because of its great fatigue strength and tolerance to high temperatures, this kind of material is advantageous in the ultrasonic atomization process. However, it appears that when exposed to the hot melt, fibers come off the plate and contaminate the powder. Due to their small size, these impurities are not expected to hinder flowability. Nonetheless, traces of carbon might negatively impact the quality of the component and the welding process by potentially causing a lack of fusion or porosity. 

### 3.6. Oxygen Content Analysis

**Figure 7:**
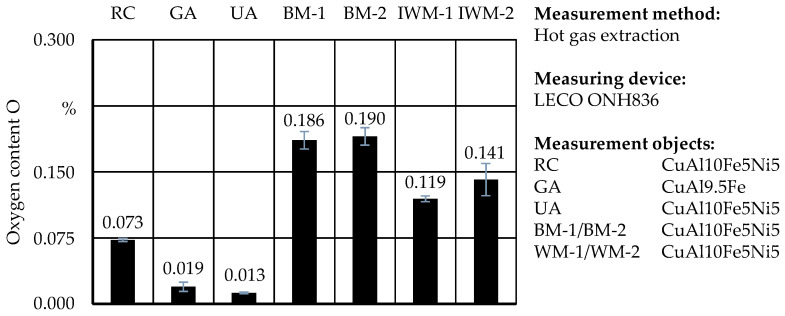
Overview of the O in % in each sample.

The oxygen content (O) plays an essential role for the quality of additively manufactured structures of metal powders and can lead to altered microstructure, increased porosity, and reduced material quality [32]. Figure 7 shows the O of the investigated samples of this study. The sieved chips contained an average amount of 0.073% O. It can be assumed that this oxidation results from the grinding process, which was carried out without cooling lubricants. Compared to the raw chips, the O increased during all comminution processes; more intense milling as with sample IWM-2 led to higher O. Other authors like Afshari et al. [9] or Lucks et al. [33] noticed very similar behaviors in their studies where the O and milling time correlated positively. Compared to the reference powder, materials from ball milling and impact whirl milling contained 5 to 10 times more oxygen. Powder UA had the lowest measured oxygen level, which could be explained by a good shielding atmosphere during atomization. While the low amount of oxygen in powder UA would most certainly not affect the deposition process and part quality, the levels in samples from ball milling and impact whirl milling would likely promote an unstable melt pool, pores, and welding fumes [30].

### 3.7. Flowability

For DED-LB and other powder-based AM processes, the flowability of the feedstock is crucial to ensure an even and controlled deposition and spreading of weld tracks and layers. In this study, flowability was assessed using the Carney flow test, the AOR, and tap and bulk density *ρ*_Sch_, from which the Hausner ratio was calculated.

**Figure 8:**
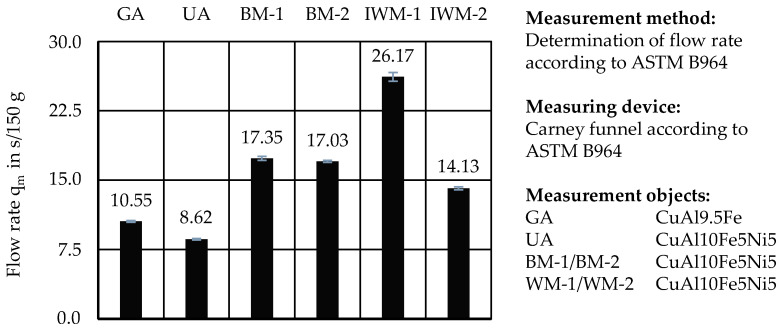
Carney flow rate measurements according to ASTM B964.

Figure 8 shows the respective flow rates q_m_ from the Carney flow test. The reference powder GA had a flow rate of q_m_ = 10.55 s/150 g. All samples from comminution showed a lower flow rate q_m_ (higher flow time t_m_). Only the ultrasonic atomized powder achieved a flow rate of q_m_ = 8.62 s/150 g and flowed better than the reference GA. Sample IWM-1 had the highest flow rate of q_m_ = 26.17 s/150 g. By comparison, the flow time t_m_ of sample IWM-2 was measured to be approximately half as long, with a value of q_m_ = 14.13 s/150 g, thus having the fastest flow from the mechanically processed materials. 

The samples of ball mill BM-1 (q_m_ = 17.35 s/150 g) and BM-2 (q_m_ = 17.03 s/150 g) did not show a significant difference in their Carney flow results.

**Figure 9:**
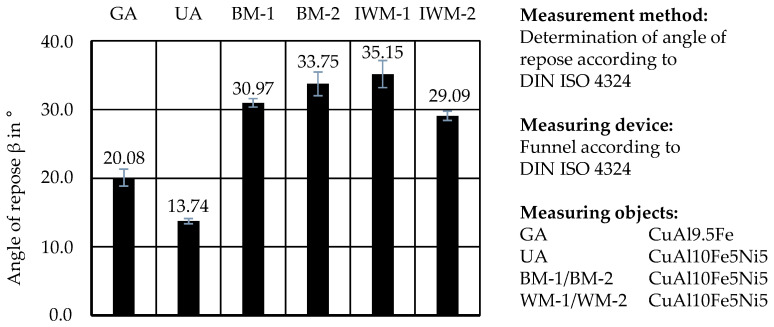
AOR measurement according to ISO 4324.

The measurements for the AOR according to ISO 4324 are displayed in Figure 9. A low AOR generally indicates a higher tendency for the powders to spread and flow. The GA powder showed an AOR of β = 20.08°. The measured angles of the mechanically prepared samples BM-2, BM-1, IWM-2, and IWM-1 were in a range from β = 29.09° to β = 35.15°. It is noticeable that sample BM-2 had a slightly shorter Carney flow time than BM-1 but had, on the other hand, a higher AOR. This could be due to the higher amount of fine particles in BM-2, which can reduce flowability due to increased inter-particle forces [5]. Powder UA achieved an AOR of β = 13.74° and exceeded the results for the gas-atomized material and any other material.

The Hausner index H, calculated as the ratio of tap to bulk density *ρ*_Sch_, is commonly used to evaluate the flowability of materials in powder analysis in various fields of research. It measures the friction conditions in moving powder masses and allows classification from excellent to poor flow behavior [34,35]. Yu and Hall [36] state that powders with a value of H < 1.25 are to be regarded as free-flowing, and those with values of H > 1.4 as non-flowing and cohesive.

**Figure 10:**
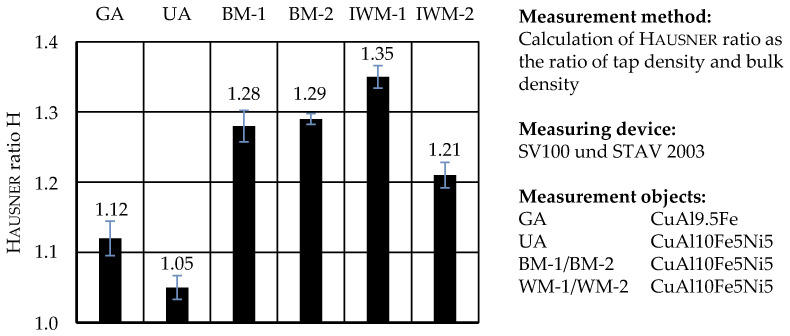
Hausner ratio H determined with the ratio of tap to bulk density *ρ*_Sch_.

The results of the Hausner ratio results are visualized in Figure 10. As with the AOR and the flow rate, the Hausner ratio diagram shows that the mechanically prepared powders from impact whirl milling and ball milling have a significantly lower flow compared to the atomized powders. The highest Hausner ratio of H = 1.35 was obtained with powder IWM-1, which is thus classified as poorly flowable [34]. Powders BM-1 and BM-2 were, as in the previous flow tests, very similar in their properties and had a passable flow of H = 1.28 and H = 1.29, respectively. From all comminuted chips, IWM-2 showed the lowest Hausner ratio of H = 1.21 and can be considered free flowing. The ultrasonic atomized powder exhibited the best result with H = 1.05 (excellent flowability).

## 4. Summary

The results of the flowability tests are consistent with the visual assessments conducted using SEM and the outcomes of dynamic image analysis. 

Ultrasonic atomization demonstrated its capability to produce feedstock with highly favorable results, surpassing the properties of the reference gas-atomized powder. The morphology was characterized by highly spherical particles with minimal agglomerates and satellites, resulting in superior flowability across all conducted flow tests.

For the ball-milled materials, the impact of increasing processing times during ball milling seems to be minimal under the current test conditions of this study; both BM-1 and BM-2 exhibited negligible differences in their PSD and morphology. However, both samples displayed elevated oxygen content (O) and suboptimal flow properties, which could lead to insufficient material feed and potential nozzle clogging during DED-LB. Implementing a multi-stage approach with varying milling ball diameters, as explored by Fullenwider et al. [11], could potentially enhance powder properties but would also increase complexity.

In the case of comminution through impact whirl milling, the increase in milling speed significantly influenced powder characteristics. IWM-1 demonstrated marginal improvement compared to the sieved raw material, retaining sharp-edged and elongated chips. In contrast, IWM-2 exhibited the most favorable properties among the mechanically comminuted powders. The pronounced rounding and size reduction of particles achieved by impact whirl milling at higher rotation speeds contributed to an advantageous morphology, resulting in improved flow properties. Consequently, this material is a promising candidate for DED-LB.

All samples from mechanical milling displayed noticeable contamination with abrasive ceramic particles from the grinding belts. While such particles were also present in material UA, their quantity was significantly lower. Few carbon fiber impurities from the sonotrode in material UA are expected to cause no significant issues.

## 5. Conclusions

This study compared feedstocks for the laser- and powder-based directed energy deposition process that were obtained from nickel aluminium bronze grinding chips through mechanical comminution and re-melting. The materials were characterized by various methods including dynamic image analysis, scanning electron microscopy, and standardized flowability tests. The following conclusions can be drawn regarding recycling grinding chips for AM feedstock:

- Ultrasonic atomization produced highly spherical and flowable powder (H = 1.05) suitable for powder-based AM processes.
- Ball milling reduced the particle size of the chips but resulted in a high O (0.19%) and an unfavorable morphology (AR_mean_ = 0.576), negatively impacting flowability (H = 1.29).
- Impact whirl milling, with sufficient rotation speeds, produced chip powders with promising properties in terms of morphology (AR_mean_ = 0.633) and flowability (H = 1.21).
- Contaminations with ceramic abrasives from the grinding process and carbon fibers from the sonotrode used in ultrasonic atomization were found in the produced powders. These contaminants impair powder quality and could lead to defects in additively manufactured parts.

In particular, the powders from ultrasonic atomization and impact whirl milling appear well-suited for the DED-LB process. Further experiments need to be performed to understand flowability under real conditions in DED-LB powder feeding units and to determine the mechanical properties of resulting AM structures from the recycled chip powders. An analysis of the environmental impact in combination with the costs of the recycled materials compared to conventional AM powder would show whether the approach also provides value on an ecological and environmental level.

## Figures and Tables

**Table 1:** Overview on materials and powders investigated and used for this study.

Abbreviation	Production Process	Additional Information/ Processing Conditions	Alloy
GA	Gas atomization	Reference, commercial AM powder	CuAl9.5Fe
SC	Sieving	Sieved with an analysis sieve (d_mesh_ = 125 µm)	CuAl10Ni5Fe5
BM-1	Ball milling	Milling time t_mill_ = 8 h	CuAl10Ni5Fe5
BM-2	Ball milling	Milling time t_mill_ = 12 h	CuAl10Ni5Fe5
IWM-1	Impact whirl milling	Rotation speed n_mill_ = 3000 rpm	CuAl10Ni5Fe5
IWM-2	Impact whirl milling	Rotation speed n_mill_ = 8000 rpm	CuAl10Ni5Fe5
UA	Ultrasonic atomization	Sonotrode frequency f = 40 kHz	CuAl10Ni5Fe5

**Table 2:** Particle size distribution for equivalent particle diameter X_A_ and aspect ratio AR.

Characteristic	GA	SC	BM-1	BM-2	IWM-1	IWM-2	UA
X_A,10_ in µm	64	81	66	54	94	72	48
X_A,50_ in µm	95	131	143	127	148	108	98
X_A,90_ in µm	135	188	254	235	212	155	174
Mean aspect ratio AR_mean_	0.843	0.558	0.558	0.576	0.543	0.633	0.935
Aspect ratio AR > 0.9 in %	43.5	2.2	1.9	2.2	1.9	4.9	82

**Table 3:** Chemical composition of the powders.

Samples	Chemical Composition of Alloys as Mass Fraction				
	Cu	Al	Fe	Ni	Mn
CC333G, DIN 1982 [19]	76.0–83.0%	8.5–10.5%	4.0–5.5%	4.0–6.0%	≤3.0%
GA	89.3%	9.3%	1.1%	-	-
SC	75.0%	10.4%	5.4%	7.7%	1.5%
IWM-1/IWM-2	78.9%	8.3%	3.8%	4.6%	0.6%
BM-1	82.31%	9.32%	2.75%	3.93%	1.70%
BM-2	82.60%	5.26%	4.33%	5.55%	2.26%
UA	75.16%	9.83%	6.76%	5.91%	2.33%

## Data Availability

Data are contained within the article.
